# Cognitive Deficits Are Associated with Frontal and Temporal Lobe White Matter Lesions in Middle-Aged Adults Living in the Community

**DOI:** 10.1371/journal.pone.0013567

**Published:** 2010-10-21

**Authors:** David Bunce, Kaarin J. Anstey, Nicolas Cherbuin, Richard Burns, Helen Christensen, Wei Wen, Perminder S. Sachdev

**Affiliations:** 1 Centre for Mental Health Research, Australian National University, Canberra, Australian Capital Territory, Australia; 2 Department of Psychology, Brunel University, London, United Kingdom; 3 Neuropsychiatric Institute, Prince of Wales Hospital, Randwick, New South Wales, Australia; 4 School of Psychiatry, University of New South Wales, Sydney, New South Wales, Australia; The University of Western Ontario, Canada

## Abstract

**Background:**

The association between brain white matter lesions and cognitive impairment in old age is well established. However, little is known about this association in midlife. As this information will inform policy for early preventative healthcare initiatives, we investigated non-periventricular frontal, temporal, parietal and occipital lobe white matter hyperintensities (WMH) in relation to cognitive function in 428 (232 women) community-dwelling adults aged 44 to 48 years.

**Results:**

Frontal white matter lesions were significantly associated with greater intraindividual RT variability in women, while temporal WMH were associated with face recognition deficits in men. Parietal and occipital lobe lesions were unrelated to cognitive performance. These findings did not differ when education and a range of health variables, including vascular risk factors, were taken into account.

**Conclusion:**

Gender differences in WMH-cognition associations are discussed, and we conclude that small vessel disease is present in midlife and has functional consequences which are generally not recognized. Preventative strategies should, therefore, begin early in life.

## Introduction

There is substantial evidence that subcortical white matter lesions are associated with cognitive deficits [for reviews see 1]–[3]. However, the majority of investigations involve persons aged over 60 years, and it is rare for research to focus on middle-aged adults. From a lifespan perspective this is a notable omission as it is important to identify when age-related cognitive deficits begin to appear, and to pinpoint factors that may explain such deficits. Not only is such work theoretically important, but practically, it may provide valuable information concerning when screening and assessment for age-related neuropathology should begin, and thereby facilitate early intervention. Here, we investigated white matter lesions and performance on a range of cognitive measures. Importantly, we focused on healthy, community-dwelling adults aged between 44 and 48 years. Our objective was to assess whether associations between white matter lesions and cognitive deficits typically reported in the over 60 s, were evident in this comparatively younger age group.

Our particular focus was on white matter hyperintensities (WMH). WMH refer to white matter lesions that appear as high signal intensities on T2-weighted MRI. Their neuropathological origins are wide-ranging and include demyelination, gliosis, destruction of axons, and eventual cavitation and infarction. As the myelinated axons within white matter form connective pathways within and between different brain structures, damage to these pathways is likely to have consequences for the efficiency of information transfer within the brain, and therefore, for cognitive function. Indeed, it is likely that white matter alterations differentially affect cognitive function depending on the brain regions involved [4].

Age, together with vascular risk factors, is one of the strongest predictors of WMH burden, and research shows WMH are associated with deficits in a range of cognitive domains including processing speed, executive function and episodic memory [1]–[3]. As noted though, the vast majority of the studies to date have focused on older ages, and it is relatively rare for WMH and cognition to be investigated in healthy adults below 50 years of age. Three recent studies that did include persons aged under 50 years [5]–[7] all demonstrated associations between white matter degradation and cognitive deficits, but did so in relatively small samples, the largest comprising 52 persons.

A major objective of the present study, therefore, was to address the paucity of research investigating associations between WMH and cognition in large population-based samples of adults aged below 50 years. Additionally, it was important to establish how far vascular risk factors account for WMH-cognitive associations in middle age. One of the aforementioned studies [7] clearly implicated vascular health as a major influence on white matter degradation, and by extension, cognitive function. Therefore, we utilized data for 428 persons aged 44 to 48 years participating in the *PATH Through Life Project*, a large-scale population-based study of age, cognition, and a range of health, biological, and individual difference variables [see 8]. On the basis of the established link between cognition and WMH, we expected cognitive deficits where WMH were present. We anticipated that such associations would be specific to the cognitive domain. Specifically, tasks drawing upon executive processes would predict frontal WMH, while memory measures would predict temporal white matter lesions. Because our focus was on non-periventricular WMH which are thought to be related to ischaemia [e.g., [9],[10]], we also anticipated that vascular risk factors would account for any WMH-cognition associations that were identified.

## Methods

### Ethics statement

All aspects of the study were approved by the Australian National University Human Research Ethics Committee. Written informed consent was obtained from all participants in the study.

### Participants

This cohort of the *PATH Through Life Project* comprised 2530 individuals aged 44–48 years who were residents of the city of Canberra and surrounding areas, and were recruited randomly through the electoral roll. Enrolment to vote is compulsory for Australian citizens. A randomly selected subsample of 656 participants was offered an MRI scan, of which 503 accepted, and 431 (85.7%) eventually completed. There were no differences in age, sex and years of education between those who had an MRI scan and those who did not (p>0.05). In the present study, WMH data for three participants were unavailable, and so the analyses reported below are based on 428 persons (232 women) with a mean age of 46.69 years (SD = 1.43). For those participants, apart from WMH reported here, MRI scans did not indicate any additional neuropathology.

### Health variables

Health histories were obtained through an interview, and included details (prevalence rates in parentheses) of cancer (n = 10; 2.3%), heart disease (n = 13; 3.0%), stroke (n = 4; 0.9%), diabetes (n = 9; 2.1%), thyroid problems (n = 19; 4.4%), and head injury (n = 67; 15.7%). All were coded 1 = Yes, 2 = No. In a minority of cases (<1.7%), missing data were coded ‘2’. This represents a conservative approach to the estimation of disease for missing data. Two readings of resting blood pressure two hours apart with participants sitting were taken by the interviewer using an Omron M4 automatic blood pressure monitor, for which they had received specific training. For present purposes, high blood pressure (BP) was defined as either mean systolic BP>140 mm Hg, or mean diastolic BP>90 mm Hg [11]. Values above those thresholds were coded ‘2’, and those below ‘1’.

### Psychomotor tasks

Simple and choice reaction tasks were administered, and for both tasks measures of intraindividual mean RT and variability were computed.

#### Simple and choice RT tasks:

These tasks were administered using a small box held with both hands, with left and right buttons at the top to be depressed by the index fingers. The front of the box had three lights: two red stimulus lights under the left and right buttons respectively and a green get-ready light in the middle beneath these. There were four blocks of 20 trials measuring simple reaction time (SRT), followed by two blocks of 20 trials measuring choice reaction time (CRT). For SRT everyone used their right hand regardless of dominance. The interval between the ‘get-ready’ light and the first light of the trial was 2.3 s for both SRT and CRT.

#### Computation of intraindividual mean RT

Means were calculated after removing outliers. This was done by firstly eliminating any values over 2000 ms. Next, means and standard deviations were calculated for each individual for each block and values were eliminated which lay outside three standard deviations for each individual. A number of very slow individuals still retained RT scores greater than 1000 ms. In a final step, these values were dropped before the final means per block were calculated for each participant. Here we present the grand mean across blocks for the respective tasks.

#### Computation of intraindividual variability

Mean absolute residuals (in ms) were calculated for each individual by averaging the deviations from regression models of RT against trial number and block number in each of the simple and choice RT series (Blocks 1–4 inclusive for simple RT, and Blocks 5–6 for choice RT). A quadratic function of trial number was also entered into the model because the decline in RT with practice is not linear. Block number was treated as categorical. These models were designed to remove both intra-block practice effects and the effect of the short rest periods between blocks, leaving residuals that measure only random variation. By contrast, simply using each person's ‘raw’ standard deviation of RT would inflate the apparent variability for participants who showed substantial improvement over the course of their trials.

This procedure is similar to that used in the cognitive aging literature more broadly [e.g., 24] and follows a precedent within the PATH Through Life Project specifically [8]. The procedure takes into account individual differences in RT when determining outliers, and the absolute cut-off at the final step ensures that intermittent unusually slow responses for this age group (for the respective tasks) are excluded. This results in a conservative measure of variability and in consequence, there is an increased likelihood that any effects found in relation to the variability measure are robust.

### Other cognitive measures

In addition to the RT tasks, a battery of cognitive tests was administered to participants. This included a *backward digit span test* from the Wechsler Memory Scale which requires participants to repeat a list of three to six words in length backwards [12]. *Immediate* and *delayed recall* were assessed using the first trial of the California Verbal Learning Test which requires participants to remember 16 shopping list items and to recall them immediately and again after a delay of twenty minutes [13]. In a *face recognition task* [14], 12 photographs of faces were presented for 45 s. After a 90 s delay, the 12 target faces were represented with 13 distracter faces. Finally a *Lexical decision making* was measured through the Spot-the-Word test [15] which comprises of 60 questions and requires participants to indicate which of two items is a valid word.

### MRI acquisition

MRI data were acquired on a 1.5 Tesla Gyroscan scanner (ACS-NT, Philips Medical Systems, Best, The Netherlands). T1-weighted 3-D structural MRI images were acquired in coronal plane using Fast Field Echo (FFE) sequence. About mid-way through this study, for reasons beyond the researchers' control, the original scanner (Scanner A) was replaced with an identical Philips scanner (Scanner B) and single channel RF headcoils. The scanning parameters were kept essentially the same. The first 163 subjects were scanned on Scanner A with TR = 8.84 ms, TE = 3.55 ms, a flip angle of 8°, matrix size = 256×256, slices 160, and field of view (FOV) 256×256 mm. Slices were contiguous with slice thickness of 1.5 mm. For the remaining 268 subjects scanned on Scanner B, the TR = 8.93 ms, TE = 3.57 ms values were slightly different in order to improve image quality, but all other parameters were exactly the same. The fluid-attenuated inversion recovery (FLAIR) sequence was the same for both scanners and acquired with TR = 11,000 ms, TE = 140 ms, TI = 2,600, number of excitations = 2, matrix size  = 256×256, and the FOV was 230×230 mm. Slice thickness was 4.0 mm with no gap between slices and in-plane spatial resolution is 0.898×0.898 mm/pixel. To ensure the reliability and compatibility of the data, we compared the subjects scanned on the two scanners on sociodemographic and imaging parameters. There were no differences on age (p = 0.377), or years of education (p = 0.588), but more women were inadvertently scanned on Scanner B than A (p = 0.003). The volumetric measures of total intracranial volume, gray matter volume, white matter volume, or cerebrospinal fluid volume obtained from two scanners did not differ significantly [16]. For the old and new scanners respectively, mean volumes were as follows: Grey matter = 0.72 vs 0.72; white matter = 0.47 vs 0.46; cerebrospinal fluid = 0.27 vs 0.27.

### Image analysis

The image analysis of WMH has been described in detail elsewhere [16]. Briefly, the FLAIR and 3D T1 structural images of the same subject were co-registered [17]; T1-weigthed structural images were segmented into three separate tissue components (grey matter, white matter, and cerebrospinal fluid [18], [19]. Nonbrain tissue was removed from both T1-weighted and co-registered FLAIR images using the brain mask transformed from the average mask originally defined in the Talairach space by inverting the deformation matrix generated from its own spatial normalization [20]; the spatial normalization transformation to produce the brain masks and white matter probability maps in the individual imaging space was inverted for the WMH detection and non-brain tissue removal; both FLAIR and T1-weighted images were intensity corrected after the removal of nonbrain tissues [19]. Finally, a parametric method [21] was adapted and applied to the initial WMH detection. Candidate WMH clusters from the brain were extracted and further investigated using a non-parametric k-nearest neighbor rule consisting of a training procedure on a small portion of the dataset and a testing procedure applied to the whole dataset. Candidate clusters were then classified into deep WMH, periventricular WMH, and false WMH clusters. This method was validated against visual ratings conducted by two independent clinicians experienced in examining MRI scans on a modified Fazekas scale [22]. The different types of white matter hyperintense signal surrounding the ventricles and in the deep white matter were rated as 0 = absence, 1 = “caps” or pencil-thin lining, 2 = smooth “halo,” 3 = irregular white matter hyperintensity extending into the deep white matter. Deep white matter hyperintense signals were rated as 0 = absence, 1 = punctate foci, 2 = beginning confluence of foci, 3 = large confluent areas. There was a strong association between visual ratings and computed WMHs volumes (r  = .823, p = 0.001).

### Missing data and statistical analyses

For missing data on a minority of cognitive variables (mean RT and variability measures for SRT and CRT tasks), values were imputed with the EM algorithm in SPSS [see 23]. Missing data frequencies before imputation were less than 3% for all variables.

Hierarchical multiple regression was used in the main analyses. WMH variables were regressed onto intracranial volume and total white matter volume at Step 1 in order to take into account individual differences in neuroanatomical structure. At Step 2, the primary effects for the cognitive variables and gender were entered into the equation. As several gender effects involving cognitive variables were evident in the bivariate correlations, at Step 3 the Gender × Cognitive variable interaction (variables were centered prior to this procedure) was entered. Due to the number of regression equations run, alpha was set conservatively at p<.01.

## Results

Descriptive statistics for the WMH variables by laterality and gender are presented in Table 1. In the light of the associations that we report with the cognitive variables below, it is important to note that a relatively small percentage of WMH were recorded across the various brain regions, and that percentages were largely similar for men and women (for further details of WMH in this sample, see 16).

**Table 1:** Descriptive data for white matter hyperintensities variables by gender^1^.

	Left Frontal		Right Frontal		Left Temp		Right Temp		Left Parietal		Right Parietal		Left Occ		Right Occ	
	M	W	M	W	M	W	M	W	M	W	M	W	M	W	M	W
% of sample	7.1	6.9	8.7	11.6	1.5	0.4	2.0	0.4	15.3	15.5	16.8	20.7	1.5	0.4	1.0	0.9
Mean vol^2^ (SD)	2.23 (12.20)	2.77 (19.06)	5.03 (30.98)	5.46 (24.31)	0.45 (3.97)	0.04 (0.59)	0.37 (2.76)	0.19 (2.86)	8.66 (37.21)	16.28 (57.95)	7.52 (26.0)	18.18 (94.59)	0.39 (3.78)	.06 (.69)	0.08 (0.91)	.81 (9.67)
Range^3^	127.5	255	346.5	244.5	45	9	27	43.5	337.5	415.5	190.5	1290	49.5	10.5	12	139.5

Notes.

1 All values computed within-gender (men = 196; women = 232).

2 Metric = mm^3^.

3 Lowest value = 0.

Temp  =  Temporal; Occ  =  Occipital; M  =  Men; W =  Women.

Bivariate correlations for all the main variables in the study are presented in Table 2. Gender differences were observed for some of the cognitive variables; women outperformed men on immediate and delayed recall, whereas the opposite was true for spot-the-word and backward digit span tasks. Apart from that expected for total white matter volume, the associations between gender and WMH were all statistically unreliable. With one exception, significant correlations indicated WMH to be associated with poorer cognitive performance. The exception concerned right temporal WMH, which were positively associated with spot-the-word scores.

**Table 2:** Bivariate correlations between biographical, cognitive and white matter variables.

	M	SD	1	2	3	4	5	6	7	8	9	10	11	12	13	14	15	16	17	18	19	20
1.Gender	−	−	−																			
2.Years Educ	14.56	2.40	−.08	−																		
3.Immed Rec	8.18	2.27	.24**	.10*	−																	
4.Del Rec	7.54	2.48	.22**	.09	.84**	−																
5.Digit Back	5.80	2.22	−.10*	.12*	.21**	.16**	−															
6.Face Recog	9.44	1.44	.09	.11*	.14**	.13**	.10*	−														
7.Lex Dec Making	51.65	4.85	−.12*	.41**	.20**	.19**	.28**	.09	−													
8.ISD SRT	0.044	.019	.06	−.07	−.06	−.05	−.14**	−05	−.09	−												
9.Mn SRT (ms)	240	42.5	.17**	−.08	−.04	−.01	−.18**	−.07	−.09	.59**	−											
10.ISD CRT	0.046	.015	.07	−.02	−.02	−.04	−.03	−.01	−.03	.37**	.29**	−										
11.Mn CRT (ms)	292	41.4	.15**	.01	.00	.02	−.08	−.02	−.06	.38**	.67**	.61**	−									
12.ICV	1449	136	−.66**	.14**	−.16**	−.13**	.12*	−.13**	.15**	.00	−.14**	−.08	−.12*	−								
13.Tot WM vol	463	55.1	−.65**	.13**	−.16**	−.12**	.11*	−.09	.11*	−.02	−.17**	−.14**	−21**	.86**	−							
14.Front WMH L	2.52	16.26	.02	.01	.01	.01	−.02	−.03	.03	.11*	.01	.12*	.06	.09	.08	−						
15.Front WMH R	5.26	27.53	.01	−.01	−.03	−.04	−.01	.09	−.02	−.01	.01	−.04	−.01	−.04	−.03	.12*	−					
16.Temp WMH L	0.23	2.72	−.08	−.05	−.06	−.09	.00	−.12*	−.11*	−.02	−.05	−.01	−.01	.11*	.09	−.01	.15**	−				
17.Temp WMH R	0.27	2.81	−.03	.05	−.10*	−.11*	.06	.01	.12*	.03	.08	.00	.04	.12*	.07	−.02	.07	.02	−			
18.Par WMH L	12.79	49.63	.08	−.07	.06	.06	.05	−.01	.02	−.06	.00	−.02	.03	−.06	−.06	.19**	.19**	.00	.05	−		
19.Par WMH R	13.30	71.95	.07	.04	−.01	.03	.00	.03	−.01	−.06	−.02	−.05	.00	−.04	−.04	.01	.10*	−.02	.01	.26**	−	
20.Occ WMH L	0.20	2.61	−.07	.01	−.03	−.06	−.04	−.01	.06	−.05	−.03	−.04	−.03	.02	.02	−.01	.12*	.13**	.35**	−.01	−.01	−
21.Occ WMH R	0.48	7.15	.05	.02	.07	.04	.06	.05	.02	−.04	−.06	−.04	−.08	−.05	−.04	−.01	−.01	−.01	.00	−.02	.00	−.01

*P<.05,

**p<.01.

ISD  =  Intraindividual variability; SRT  =  Simple RT; CRT  =  Choice RT; ICV  =  Intracranial volume; WMH  =  white matter hyperintensities (ICV and WMH  =  mm^3^); Gender, 1 = male, 2 = female.

The results of the hierarchical regressions are presented in Table 3. Three features of the findings should be highlighted. First, where associations with cognitive variables exist, they involve the frontal and temporal lobes, but not the parietal and occipital lobes. Second, associations are predominantly with left hemisphere WMH volumes. In addition, greater left frontal lesioning was associated with higher intraindividual variability in choice RT, and greater temporal WMH burden was associated with poorer Spot-the-Word scores. However, both of these effects were modified by significant Gender × Cognitive variable interactions. Additionally, that interaction was significant for face recognition, although the primary effects for that regression were statistically unreliable.

**Table 3:** White matter hyperintensities regressed on cognitive variables.

	Left Frontal	Right Frontal	Left Temp	Right Temp	Left Parietal	Right Parietal	Left Occ	Right Occ
*Step*	Beta	Beta	Beta	Beta	Beta	Beta	Beta	Beta
1^a^.WM vol	.03	.02	−.02	−.13	−.04	−.02	.03	.04
IC vol	.07	−.06	.13	.23	−.02	−.02	−.01	−.09
2^b^. Immed Rec (IR)	.01	−.03	−.04	−.10	.04	−.03	−.01	.06
Gender	.14	−.03	.00	.09	.06	.10	−.09	.02
3^c^. Gender × IR	−.12	.05	.03	.06	−.03	−.03	.03	.06
2^b^. Del Rec (DR)	.00	−.04	−.07	−.11	.04	.01	−.05	.03
Gender	.14	−.02	.01	.09	.06	.09	−.09	.03
3^c^. Gender × DR	−.11	.09	.06	.03	−.02	−.01	.07	.02
2^b^. Digit Back (DB)	−.03	.00	−.01	.05	.06	.01	−.04	.07
Gender	.14	−.03	−.01	.06	.07	.09	−.10	.04
3^c^. Gender × DB	−.05	.02	.00	.05	.02	.00	.04	.06
2^b^. Face Rec (FR)	−.03	.08	−.10	.03	−.01	.02	−.01	.04
Gender	.14	−.03	−.01	.06	.07	.09	−.10	.03
3^c^. Gender × FR	−.04	−.03	.14*	−.04	−.01	.02	.01	.03
2^b^. Lexical DM (LDM)	.02	−.01	−.13*	.10	.03	.00	.05	.03
Gender	.14	−.03	−.02	.07	.07	.09	−.09	.04
3^c^. Gender × LDM	−.02	.05	.13*	−.02	−.01	.00	−.07	.04
2^b^. ISD SRT (ISRT)	.11	−.01	−.02	.02	−.06	−.06	−.04	−.04
Gender	.14	−.03	−.01	.06	.08	.10	−.09	.04
3^c^. Gender × ISRT	.09	.01	.02	−.06	−.04	−.03	.05	−.03
2^b^. MSRT	.02	.01	−.03	.08	−.02	−.03	−.02	−.07
Gender	.14	−.03	−.01	.05	.07	.09	−.09	.04
3^c^. Gender × MSRT	.08	.06	.03	−.14*	−.05	−.02	.05	−.05
2^b^. ISD CRT (ICRT)	.13*	−.04	.00	.01	−.02	−.05	−.04	−.05
Gender	.14	−.03	−.01	.06	.07	.10	−.10	.03
3^c^. Gender × ICRT	.16*	.05	−.02	−.02	−.05	−.03	.05	−.03
2^b^. MCRT	.07	−.01	.00	.04	.02	−.01	−.02	−.08
Gender	.14	−.03	−.01	.06	.07	.09	−.09	.04
3^c^. Gender × MCRT	.09	.02	.00	−.08	.02	−.01	.03	−.07

Notes:

*p<.01; a  =  df 2, 425; b  =  df 2, 423; c  =  df 1,422.

WM vol  =  White matter volume; IC vol  =  Intracranial volume; ISD  =  Intraindividual standard deviation; MSRT  =  Mean Simple RT; MCRT  =  Mean Choice RT; Temp  =  Temporal; Occ  =  Occipital.

**Figure 1:**
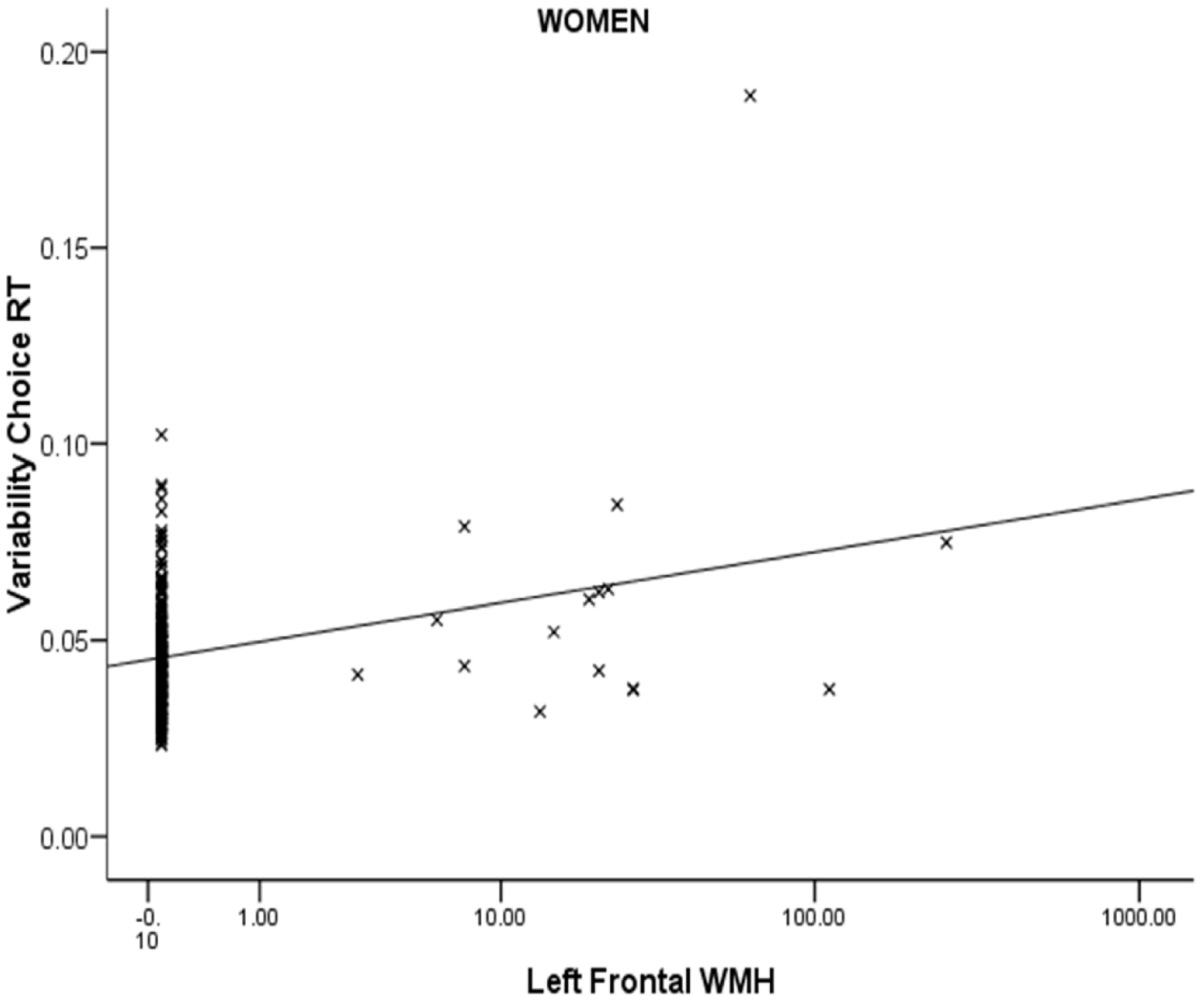
Left frontal white matter hyperintensities and choice RT intraindividual variability (women only).

Therefore, where significant Gender × Cognition interactions were found, regressions were rerun for men and women separately. With one exception, all interactions stemmed from stronger effects in men. The exception was the association between left frontal WMH and variability in the choice RT task. Although this was the only primary effect to attain significance across the whole sample (14 men and 16 women exhibited left frontal WMH), when the regression was rerun for men, it was statistically unreliable. However, for women, that regression was significant, df = 1,228, beta = .23, p<.001, indicating the association between WMH and variability to be stronger in this group (see Figure 1). Consideration of Figure 1 indicates that an outlier may have influenced this effect. Therefore, we reran the regression having removed this extreme value. The association remained positive and significant (beta = .13, p<.05) as in the original analysis.

When the remaining regressions producing significant Gender × Cognitive variable interactions were rerun within gender, for women, all associations were nonsignificant. By contrast, however, for men, significant associations indicated that greater WMH burden was associated with poorer cognitive performance: Left temporal lobe and face recognition, df = 1,192, beta = −.17, p = .014; left temporal lobe and Spot-the-Word scores, df = 1,192, beta = −.19, p = .008; right temporal lobe and simple mean RT, df = 1,192, beta = .24, p<.001.

We then repeated the analyses taking years of education into account. This had little bearing on the initial regression findings. Importantly, as health status, and in particular vascular risk factors, may influence white matter-cognition relations, we then statistically controlled (by entering health factors individually at Step 1 of the hierarchical multiple regression) for histories of cancer, thyroid problems, head injury, diabetes, stroke, heart disease, and high blood pressure (blood pressure variables were entered as both dichotomous and continuous variables). Notably, none of these variables altered our original findings.

Finally, WMH data are highly skewed and this, together with outliers, may have influenced the findings. In order to reduce the influence of these sources of variance, we reran the main analyses using sequential logistic regression having recoded the WMH variables (0 = no WMH, and 1 = >0 WMH). For left frontal WMH and variability in the CRT task, entry of variability significantly raised the probability of the presence of WMH, B = 0.38, OR = 1.46, CI = 1.09–1.96, p = .011. As subsequent entry of the Gender × Variability interaction term approached significance at conventional levels (p = .068), we ran logistic regression within men and women. For men, the regression was nonsignificant. For women however, greater variability was associated with the presence of left frontal WMH, B = 0.61, OR = 1.83, CI = 1.19–2.82, p = .006. These findings are consistent with the earlier linear regressions.

For left temporal WMH and face recognition, the findings were similar to the earlier analyses. When face recognition was entered into the equation, statistics indicated the presence of left temporal WMH were associated with poorer face recognition B = −0.69, OR = 0.50, CI = 0.26–0.97, p = .039. Although entry of the Gender × Face recognition interaction was nonsignificant, further analysis revealed the trend was stronger in men. By contrast, logistic regressions examining left temporal WMH and spot-the-word, and right temporal WMH and simple mean RT, were inconsistent with the earlier analyses.

## Discussion

This is one of the first investigations to focus on WMH and cognitive function in a large population-based sample of middle-aged adults. Several important findings suggested the possible presence of neuropathology in this relatively young and independently functioning group of 44 to 48 year olds living in the community. First, frontal lobe white matter lesions were associated with increased intraindividual variability, and temporal lobe WMH with deficits in face recognition. Second, these findings were left-lateralized, and the frontal lobe associations stronger in women, while the temporal lobe associations were stronger in men. Finally, statistically controlling for a range of health variables, including vascular risk factors, made no difference to those findings.

That WMH were associated with cognitive deficits was not in itself unusual, and is consistent with findings elsewhere [see 1]–[3]. What is of note, however, is that this association was evident in a community-based sample of functioning persons in midlife. Although the effect sizes were relatively small, the findings are consistent with work elsewhere that has found an association between white matter integrity and cognition in persons less than 50 years of age [5]–[7]. From a lifespan perspective, these findings are important as they add to evidence that the deleterious effects of neurobiological disturbance may manifest at an earlier age than is suggested by the broader literature. Not only is this of note theoretically as it points to a possible neuropathological basis for cognitive decline in middle age, but also practically, as it suggests that preventative programs and early intervention may benefit community-dwelling adults in their 40 s and upwards.

The results were selective in that left frontal lobe lesions were associated with within-person variability, while left temporal WMH were associated with spot-the-word and face recognition performance. The former finding is in line with the proposition that intraindividual variability indexes neurobiological disturbance [e.g., 24] and executive and attentional control mechanisms supported by the frontal cortex [e.g., [25]–[27]]. This finding builds upon our earlier work showing frontal lesions to predict within-person variability in adults aged 60 to 64 years [28], and the left lateralization of the association is consistent with functional imaging work showing an association between left middle frontal (BA 46) activity and within-person variability [29]. Together, these studies suggest the neural correlates of intraindividual variability in young and middle-aged persons to include the left dorsolateral prefrontal cortex.

It is of note that, although the association between left frontal WMH and intraindividual variability was stronger in women, the primary effect was also significant for this regression (see Table 3) indicating the trend to be present in men too (although this effect was nonsignificant when tested in men alone). One factor that may have contributed to the stronger effect in women is the likelihood that individuals in this age group were perimenopausal, and hormonal factors may have influenced the strength of this association. Indeed, there is evidence that estrogen may moderate variability over time in women [30].

It is important to emphasize that while the measure of within-person variability was sensitive to WMH presence, the alternative measure of central tendency (mean RT) for the same choice RT task, was not. This finding adds to work showing a dissociation between measures of mean RT and within-person variability from the same task, with the latter variable being sensitive to possible neuropathology [28] and mild psychopathology [31], [32]. Given the apparent sensitivity of this measure to subtle effects when other cognitive measures are not, and evidence that intraindividual variability predicts conversion to mild cognitive impairment over several years [33], it is possible that these measures may serve as a valuable “early warning” screening tool in community and healthcare settings.

The association between left temporal WMH and spot-the-word and recognition performance was in line with, respectively, work implicating that lobe in the processing of nouns [34], and lesion studies showing that damage to the amygdala impairs face and emotion recognition [e.g., 35]. Also, right temporal lobe WMH were associated with slower responding in the simple RT task. However, some caution is appropriate in interpreting these temporal lobe findings as they stemmed from three and four men for left and right temporal lobe WMH respectively. Moreover, for spot-the-word and mean simple RT, the logistic regression failed to confirm earlier associations found using linear regression, suggesting that the skewed distribution and outlying WMH values may have influenced the initial finding. As both lexical decision making and perceptual speed measures may provide valuable insights into possible neuropathology in midlife, it is important that further work investigates these associations in middle-aged samples.

It is important to note that statistically controlling for a range of health variables, including histories of cancer, heart disease, thyroid problems, diabetes, stroke, head injury, and high blood pressure, had no bearing on the findings. The analyses controlling for vascular risk factors were of particular note as evidence suggests that non-periventricular WMH are associated with ischaemia [e.g., [9],[10]], and the finding is contrary to that reported by Raz and colleagues [7]. Although the prevalence of health risk factors in this relatively young sample may have been too low to statistically account for the WMH-cognition associations, the present findings suggest that non-vascular influences, such as age and genetics, affect WMH-cognition associations in this relatively young and predominantly healthy group. Additionally, we cannot rule out the possibility that these adults were in the preclinical phase of, as yet, undetected neurological disorder. So as to inform healthcare intervention strategy and policy, it is clearly important that further research investigates WMH-cognition relations in midlife samples, and delineates between age and health factors in accounting for associations where they are found. Importantly, longitudinal research is required that examines midlife status on a range of health, cognitive and neuropathological (e.g., WMH) markers in relation to long-term mental health outcomes, including cognitive impairment and dementia.

There are a number of limitations to the present research that should be acknowledged. First, the study was cross-sectional, and we are therefore unable to give any indication of causality. Moreover, the use of a narrow cohort design allows individual differences in characteristics such as WMH to be investigated without the confounding of age differences. However, this means that we cannot generalise our results beyond the ages of 44 to 48 years. Second, at present we do not have any information concerning the future neurological status of participants. Planned long-term follow-ups in this group will provide valuable information on how far the present findings represent the early manifestation of eventual age-related neurological conditions. Finally, due to the young age of the sample, there were relatively few participants with significant white matter lesion load. Therefore, despite the sample being arguably the largest to investigate WMH and cognition in persons in their mid−40 s, even larger samples are required to enable detailed evaluation of the small group of individuals who demonstrate significant pathology in this age group.

To conclude, the finding that cognitive deficits were associated with non-periventricular WMH in a community sample aged 44 to 48 years having taken into account a range of health variables has important implications. From a lifespan perspective, the findings suggest that cognitive deficits may have a neuropathological basis that manifests in some individuals during middle age. From a healthcare perspective this underlines the view that population-based preventative strategies should start in early adulthood and not wait until mid or later life. Not only are the costs of such initiatives likely to be offset by long-term healthcare savings, but also by associated benefits to the quality of life and extended independence of vulnerable persons living in the community.

## References

[1] Gunning-Dixon FM, Brickman AM, Cheng JC, Alexopoulos GS (2009). Aging of cerebral white matter: a review of MRI findings.. Int J Geriatr Psychiatry.

[2] Gunning-Dixon FM, Raz N (2000). The cognitive correlates of white matter abnormalities in normal aging: a quantitative review.. Neuropsychology.

[3] Raz N, Rodrigue KM (2006). Differential aging of the brain: patterns, cognitive correlates and modifiers.. Neurosci Biobehav Rev.

[4] Sullivan EV, Pfefferbaum A (2006). Diffusion tensor imaging and aging.. Neurosci Biobehav Rev.

[5] Kennedy KM, Raz N (2009). Aging white matter and cognition: differential effects of regional variations in diffusion properties on memory, executive functions, and speed.. Neuropsychologia.

[6] Madden DJ, Spaniol J, Costello MC, Bucur B, White LE (2009). Cerebral white matter integrity mediates adult age differences in cognitive performance.. J Cogn Neurosci.

[7] Raz N, Rodrigue KM, Kennedy KM, Acker JD (2007). Vascular health and longitudinal changes in brain and cognition in middle-aged and older adults.. Neuropsychology.

[8] Anstey KJ, Dear K, Christensen H, Jorm AF (2005). Biomarkers, health, lifestyle, and demographic variables as correlates of reaction time performance in early, middle, and late adulthood.. Quarterly Journal of Experimental Psychology.

[9] Fazekas F, Kleinert R, Offenbacher H, Schmidt R, Kleinert G (1993). Pathologic correlates of incidental MRI white matter signal hyperintensities.. Neurology.

[10] Wen W, Sachdev PS (2004). Extent and distribution of white matter hyperintensities in stroke patients: the Sydney Stroke Study.. Stroke.

[11] Joint National Committee on Prevention, Detection, Evaluation, and Treatment of High Blood Pressure (1997). The sixth report of the Joint National Committee on Prevention, Detection, Evaluation, and Treatment of High Blood Pressure.. Archives of Internal Medicine.

[12] Wechsler D (1991). Wechsler memory scale - revised manual..

[13] Delis DC, Kramer JH, Kaplan E, Ober BA (1987). California Verbal Learning Test..

[14] Crane J, Milner B (2002). Do I know you? Face perception and memory in patients with selective amygdalo-hippocampectomy.. Neuropsychologia.

[15] Baddeley A, Emslie H, Nimmo-Smith I (1992). The Spot-The-Word Test..

[16] Wen W, Sachdev PS, Li JJ, Chen X, Anstey KJ (2009). White matter hyperintensities in the forties: their prevalence and topography in an epidemiological sample aged 44-48.. Hum Brain Mapp.

[17] Wells WM, Viola P, Atsumi H, Nakajima S, Kikinis R (1996). Multi-modal volume registration by maximization of mutual information.. Med Image Anal.

[18] Ashburner J, Friston K (1997). Multimodal image coregistration and partitioning-a unified framework.. Neuroimage.

[19] Ashburner J, Friston KJ (2005). Unified segmentation.. Neuroimage.

[20] Ashburner J, Friston KJ (1999). Nonlinear spatial normalization using basis functions.. Hum Brain Mapp.

[21] Wen W, Sachdev P (2004). The topography of white matter hyperintensities on brain MRI in healthy 60- to 64-year-old individuals.. Neuroimage.

[22] Fazekas F, Chawluk JB, Alavi A, Hurtig HI, Zimmerman RA (1987). MR signal abnormalities at 1.5 T in Alzheimer's dementia and normal aging.. AJR Am J Roentgenol.

[23] Schafer JL, Graham JW (2002). Missing data: our view of the state of the art.. Psychol Methods.

[24] Hultsch DF, Strauss E, Hunter MA, MacDonald WS, Craik FIM, Salthouse TA (2008). Intraindividual variability, cognition and aging.. The handbook of aging and cognition, 3rd ed.

[25] Bunce DJ, Warr PB, Cochrane T (1993). Blocks in choice responding as a function of age and physical fitness.. Psychol Aging.

[26] Bunce D, MacDonald SW, Hultsch DF (2004). Inconsistency in serial choice decision and motor reaction times dissociate in younger and older adults.. Brain Cogn.

[27] West R, Murphy KJ, Armilio ML, Craik FI, Stuss DT (2002). Lapses of intention and performance variability reveal age-related increases in fluctuations of executive control.. Brain Cogn.

[28] Bunce D, Anstey KJ, Christensen H, Dear K, Wen W, Sachdev P (2007). White matter hyperintensities and within-person variability in community-dwelling adults aged 60–64 years.. Neuropsychologia.

[29] Bellgrove MA, Hester R, Garavan H (2004). The functional neuroanatomical correlates of response variability: evidence from a response inhibition task.. Neuropsychologia.

[30] Wegesin DJ, Stern Y (2004). Inter- and intraindividual variability in recognition memory: effects of aging and estrogen use.. Neuropsychology.

[31] Bunce D, Handley R, Gaines SO (2008). Depression, anxiety, and within-person variability in adults aged 18 to 85 years.. Psychol Aging.

[32] Bunce D, Tzur M, Ramchurn A, Gain F, Bond FW (2008). Mental health and cognitive function in adults aged 18 to 92 years.. J Gerontol B Psychol Sci Soc Sci.

[33] Cherbuin N, Sachdev P, Anstey KJ Neuropsychological predictors of transition from healthy cognitive aging to mild cognitive impairment: the PATH Through Life Study.. American Journal of Geriatric Psychiatry.

[34] Perani D, Cappa SF, Schnur T, Tettamanti M, Collina S (1999). The neural correlates of verb and noun processing: A PET study.. Brain.

[35] Adolphs R, Tranel D, Damasio H, Damasio A (1994). Impaired recognition of emotion in facial expressions following bilateral damage to the human amygdala.. Nature.

